# Effect of Air Pollution on the Basal DNA Damage of Mother–Newborn Couples of México City

**DOI:** 10.3390/toxics11090766

**Published:** 2023-09-09

**Authors:** Mahara Valverde, Adriana Granados, Mirta Milić, Marcello Ceppi, Leticia Sollano, Stefano Bonassi, Emilio Rojas

**Affiliations:** 1 Laboratorio de Genotoxicología, Instituto de Investigaciones Biomédicas, U.N.A.M., Mexico City 04510, Mexico; mahara@iibiomedicas.unam.mx (M.V.); adrigr@yahoo.com (A.G.); 2 Mutagenesis Unit, Institute for Medical Research and Occupational Health, Ksaverska Cesta 2, 10 001 Zagreb, Croatia; mmilic@imi.hr; 3 Clinical Epidemiology Unit, IRCCS Ospedale Policlinico San Martino, 16132 Genova, Italy; marcello.ceppi@hsanmartino.it; 4 Centro Médico Nacional 20 de Noviembre, I.S.S.S.T.E, Mexico City 03229, Mexico; letuskids@prodigy.net.mx; 5 Unit of Clinical and Molecular Epidemiology, IRCCS San Raffaele, 00166 Rome, Italy; 6 Department of Human Sciences and Quality of Life Promotion, San Raffaele University, 00166 Rome, Italy

**Keywords:** comet assay, human biomonitoring, NOx, PM10, tail length

## Abstract

Environmental pollution of megacities can cause early biological damage such as DNA strand breaks and micronuclei formation. Comet assay tail length (TL) reflects exposure in the uterus to high levels of air pollution, primarily ozone and air particles (PM_10_), including mothers’ smoking habits during pregnancy, conditions which can lead to low birth weight. In this biomonitoring study, we evaluated basal DNA damage in the cord blood cells of newborn children from Mexico City. We found a correlation between DNA damage in mothers and their newborns, including various parameters of environmental exposure and complications during pregnancy, particularly respiratory difficulties, malformations, obstetric trauma, neuropathies, and nutritional deficiencies. Mothers living in the southern part of the city showed double DNA damage compared to those living in the northern part (TL 8.64 μm vs. 4.18 μm, *p* < 0.05). Additionally, mothers’ DNA damage correlates with exposure to NOx (range 0.77–1.52 ppm) and PM_10_ (range 58.32–75.89 μg/m^3^), as well maternal age >29. These results highlight the sensitivity of the comet assay in identifying differential in utero exposure for newborns whose mothers were exposed during pregnancy. They also suggest the importance of antioxidants during pregnancy and the role of the placental barrier in protecting the newborn from the DNA-damaging effects of oxidative pollution.

## 1. Introduction

Air pollution is characterized by a complex mixture of gases and particles whose sources and composition are closely related. Particulate matter (PM), ozone (O_3_), nitrogen oxides (NO, NO_2_), sulfur oxides (SO, SO_2_), and carbon monoxide (CO) are the main pollutants used to quantify exposure to air pollution. Today, there is a large body of literature linking the presence of these contaminants with adverse health effects [1].

The metropolitan area of Mexico City is a heavily polluted area with a wide variety of pollutants, among which ozone and airborne particles play a significant role [2]. Due to the ring of mountains encircling the city, winds in the metropolitan area of Mexico City follow specific routes, leading to a non-uniform distribution of pollutants across different areas. Mexico City has one of the highest concentrations of children in the world, with around 500,000 registered births each year. Several studies in recent years evaluated both the mortality and morbidity associated with the effect of pollution in Mexico [3,4,5]. The high concentration of pollutants, mainly particulate matter and to a lesser extent ozone, has been associated with an increase in different diseases such as neurodegeneration and with excess mortality from cardiovascular and respiratory failure [6,7,8,9]. It is important to point out that these results are consistent with reports from other highly polluted cities [9,10,11,12,13,14].

The symptoms associated with environmental pollution, most investigated in Mexico, include impaired lung function, respiratory symptoms, inflammation, altered heart rate, and asthma [15,16,17,18,19,20], information consistent with that of other polluted cities in the world [21,22].

With regard to environmentally induced diseases, molecular biomarkers play a key role in understanding the relationships between exposure to genotoxic chemicals and the development of chronic diseases, as well as in the identification of subgroups and individuals with higher risk. Among these biomarkers, in the last decades [22,23], single-cell gel electrophoresis or comet assay, since its original version proposed by Singh et al. in 1988 [24], has become a preferred test for DNA damage assessment in human biomonitoring studies, with its alkaline version allowing the detection of different types of DNA damage, such as single-strand breaks (SSB), sites alkali labile (apurinic/apyrimidinic (AP) sites), crosslinks, and incomplete DNA repair sites in single cells. More recently, standardized assay protocols have been published that also highlight its use in biomonitoring studies [25].

In Mexico City, several studies evaluated the genotoxic effect of air pollution using the comet assay [26,27,28,29,30]. Most of these studies found that inhabitants of Mexico City present more damage than those living outside the urban area and associated this damage to oxidative stress [26,27,28]. Two studies measuring DNA damage in peripheral blood lymphocytes and exfoliated epithelial cells from nasal mucosa and tears found that residents living in the southern part of the city show a higher incidence of damage [29,30].

In the present study, the comet assay was used to assess baseline DNA damage in cord blood cells from newborns and to correlate it to the damage in peripheral blood lymphocytes from their mothers. Given that the mothers lived during pregnancy in different zones of the metropolitan area of Mexico City with variable concentrations of contaminants, the objective of the study was to investigate the potential effect of this non-uniform distribution of the contaminant on the genomic integrity of the mother–newborn couples.

## 2. Materials and Methods

If not mentioned otherwise, all the chemicals and materials were from Sigma Aldrich, Burlington, VT, USA.

### 2.1. Study Population

The study population was composed of 104 newborns: 64 born in the Centro Médico Nacional 20 de Noviembre, located in the Southwest zone of the city, and 40 born in the Hospital Gustavo Baz Prada, located in the Northeast zone. Thirty-nine mothers from the first group and all 40 mothers from the second group were also included. All the mothers involved in the study have the same socio-economic status, and their place of residence is in an urban environment (Mexico City). Heparinized cord blood samples and maternal venous blood samples were kindly provided by Dr. Fernando Escobedo, Dr. Patricia Birott and Dr. Luis Amaro.

The ethics committees of both hospitals approved the protocols for sample collection and analysis from both cord blood and maternal venous samples together with the questionnaires, in accordance with the declaration of Helsinki (HGBP/057/0998). Informed consent was obtained from all the subjects (from mothers and mothers signed in the name of their newborns) involved in the study. Questionnaires were used to gather personal information, lifestyle details, and medical history from the mothers.

Newborns’ health parameters, including weight, length, and Apgar scores at one and five minutes from the birth, were also recorded. Low birth weight was defined according to the national standards curves (<2500 g). Additionally, newborns‘ complications such as respiratory difficulties, malformations, obstetric trauma, neuropathies, hypoxia, and nutritional deficiencies were also documented.

### 2.2. Pollution Data

The Automatic Atmospheric Monitoring Network or The Red Automatizada de Monitoreo Ambiental (R.A.M.A.) provided pollutant data for Mexico City during the sampling year. The website network of R.A.M.A. has been organized as an annual database that contains information on the concentrations of pollutants that have been recorded every hour since 1986 (http://www.aire.cdmx.gob.mx/, accessed on 17 September 2017). For analysis purposes, the Mexico City metropolitan area was divided into zones, namely northeast (NE), northwest (NW), southeast (SE), and southwest (SW). The average concentrations of different pollutants (ozone, particulate matter-PM_10_, sulfur dioxide, carbon monoxide and dioxide, and nitrogen oxide and dioxide) were obtained for each mother during their gestation period by means of the R.A.M.A database reports (http://www.aire.cdmx.gob.mx, accessed on 17 September 2017) accessed on 17 September 2017. It should be noted that in recent years, ozone concentrations were indistinctly higher in the southern part of the city. Therefore, the metropolitan area was also divided into north (N) and south (S) zones to calculate the concentrations of pollutants in these zones. Alternatively, separate analyses were performed considering the area of residence of the mothers, based on two or four subgroups.

### 2.3. Alkaline Comet Assay

The comet assay was performed within a maximum of 12 h after the sample collection, and the protocol used, with some modifications, was as described by Singh et al. [24] and Tice et al. [31]. Briefly, 10 μL of whole blood was mixed with 75 μL of 0.5% low melting point (LMP) agarose, and the mixture was spread on microscopic slides previously covered by a layer of 0.5% normal melting point agarose. After solidification at 4 °C, another layer of 0.5% LMP agarose was added and solidified. Lysis was carried out for at least one hour at 4 °C (2.5 M NaCl, 0.1 M EDTA, 10 mM Tris, pH10, freshly added 1% Triton X-100, and 10% DMSO). Alkali unwinding was performed for 20 min (0.3 M NaOH, 1 mM Na2EDTA, pH > 13) and electrophoresis was conducted for 20 min in the same buffer at 0.8 V/cm under the dim light. The slides were neutralized with 0.4 M Tris (pH 7.5) and then stained by adding 20 μL of ethidium bromide (20 μg/mL). Fifty nucleoids per slide were evaluated on two slides per individual. The comet’s head and tail were defined as described previously [30]. The nucleoids were scored, determining DNA migrations (microns, μm) in the comet tail (comet tail length, TL) as well as the comet head with an ocular micrometer.

### 2.4. Statistical Analysis

Descriptive statistics concerning mother–newborn couples are reported in Table 1. They include quantitative and qualitative parameters of the mother’s pregnancy and newborns such as maternal age, zone of residence, exposure to air pollution, and health status, and DNA damage was reported as mean ± standard deviation (SD) or percentage for continuous or discrete variables, respectively. Then, univariate analysis was carried out to evaluate the effect of each variable on the level of DNA damage, measured as tail length (TL). All variables were categorized according to clinical parameters whenever possible, e.g., birth weight < 2500 g, or—as in the case of pollutants—using tertiles.

The difference in the level of DNA damage was measured for each variable with the Chi-square test for qualitative variables and with the Student’s *t*-test for continuous variables. Logarithmic transformation of data was applied to normalize the distributions of variables, which showed a significant departure from normality according to the Kolmogorov–Smirnov test.

Lognormal models were applied to study variables associated with TL in mothers and newborns. The mean ratio (MR), together with its asymptotic 95% confidence intervals (95% CI), was computed for all variables included in the final models (only variables significantly associated to DNA damage were reported in the tables). The following variables were included in all models as potential confounders: maternal age (years); multiple births; number of pregnancies; number of abortions; infections during pregnancy; use of medications during pregnancy; smoking during pregnancy; weeks of gestational age; birth weight (%); Apgar score at 1 min; Apgar score at 5 min; area of residence; NOx (ppm); PM_10_ (μg/m^3^); and ozone at delivery (ppm). Due to small numbers, a forward variable selection procedure was adopted, iteratively adding variables one at a time, selecting the variable that improves the model fit the most at each step. No interaction was found to be significantly associated to the outcome. To reduce the presence of multicollinearity, a correlation analysis was performed to identify and select variables that were highly correlated. The critical limit for significance was set at *p* < 0.05. The statistical software used for all analyses was SPSS (SPSS for Windows, 28.0).

## 3. Results

The mean age of mothers (± SD) living in Mexico City that delivered a newborn and were included in the study was 28.49 ± 6.34 years, with a mean gestation time of 37.1 ± 3.36 weeks. Approximately 70% of them had previous pregnancies, did not have any history of abortion, were not diagnosed with any infections, and did not receive any medical treatment during pregnancy. Six mothers (13.3%) reported smoking during pregnancy (Table 1).

Most newborns (76.7%) had a regular weight at birth (≥2500 g), and their condition was good at the 1st and the 5th minute after birth (Apgar score), with a mean score of 7.99/10.0 at 1st min and 8.81/10.0 at 5th min. As for the place of residence, mothers lived in all parts of Mexico City (Table 1), with a higher concentration in the northeast area (47.9%).

The pollution level in the city—as expected—was rather high, with the mean 9-months average concentration of air pollutants exceeding most international standards for urban air pollution, i.e., nitrogen oxide (NOx) 1.32 ppm, PM_10_ 69.25 μg/m^3^, and ozone 0.81 ppm (Table 2). Sulfur dioxide, carbon monoxide and dioxide, and nitrogen dioxide did not show variation in concentration, which is of interest for this study (data not shown).

**Figure 1:**
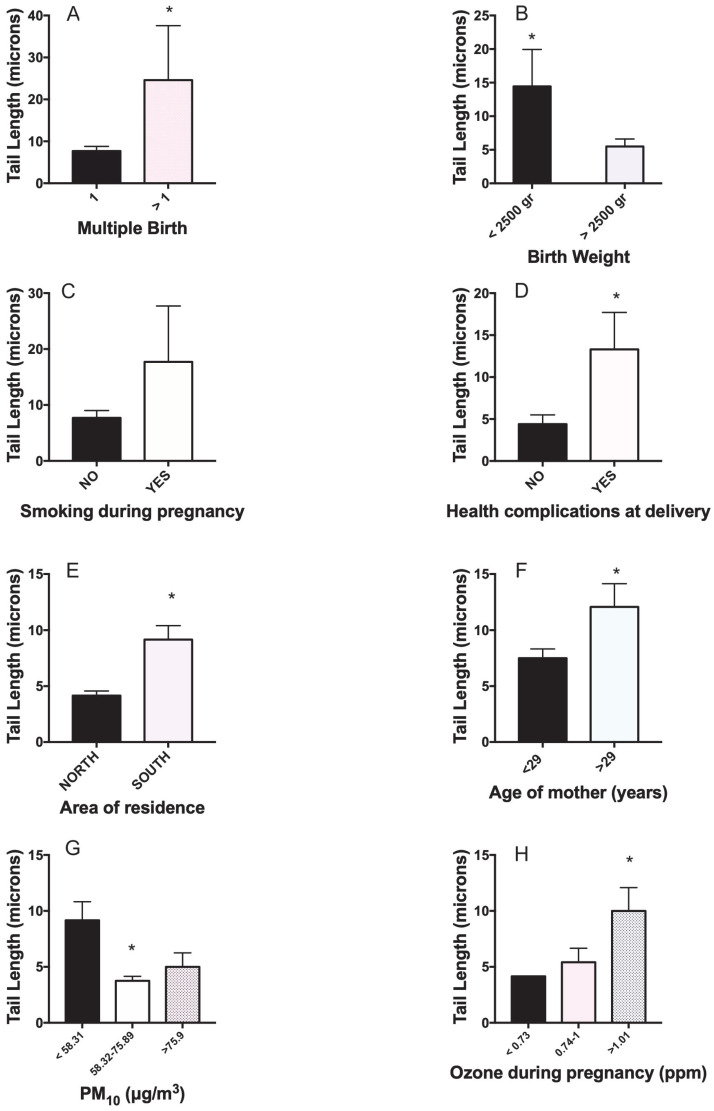
Univariate analysis linking DNA damage (TL) in resident mothers of Mexico City to main predictive variables. (**A**) Multiple births, (**B**) birth weight, (**C**) smoking habit during pregnancy, (**D**) health complications at delivery, (**E**) area of residence, (**F**) age of mother, (**G**) PM_10_ concentration, (**H**) ozone at delivery day. * *p* < 0.05.

To evaluate the influence of study parameters on the level of DNA damage in mothers and their newborns, univariate analysis was preliminarily conducted. Significantly higher damage was found in those mothers delivering multiple births, having products of less than 2500 gr, or experiencing a health complication at delivery (Figure 1A,B,D). Other parameters significantly affecting DNA damage were residence in the southern part of Mexico City, delivery after 29 years of age, being exposed to the highest tertile of ozone at delivery day, and unexpectedly being exposed to PM_10_ in a range of 58.32–75.89 μg/m^3^ (Figure 1E–H).

**Figure 2:**
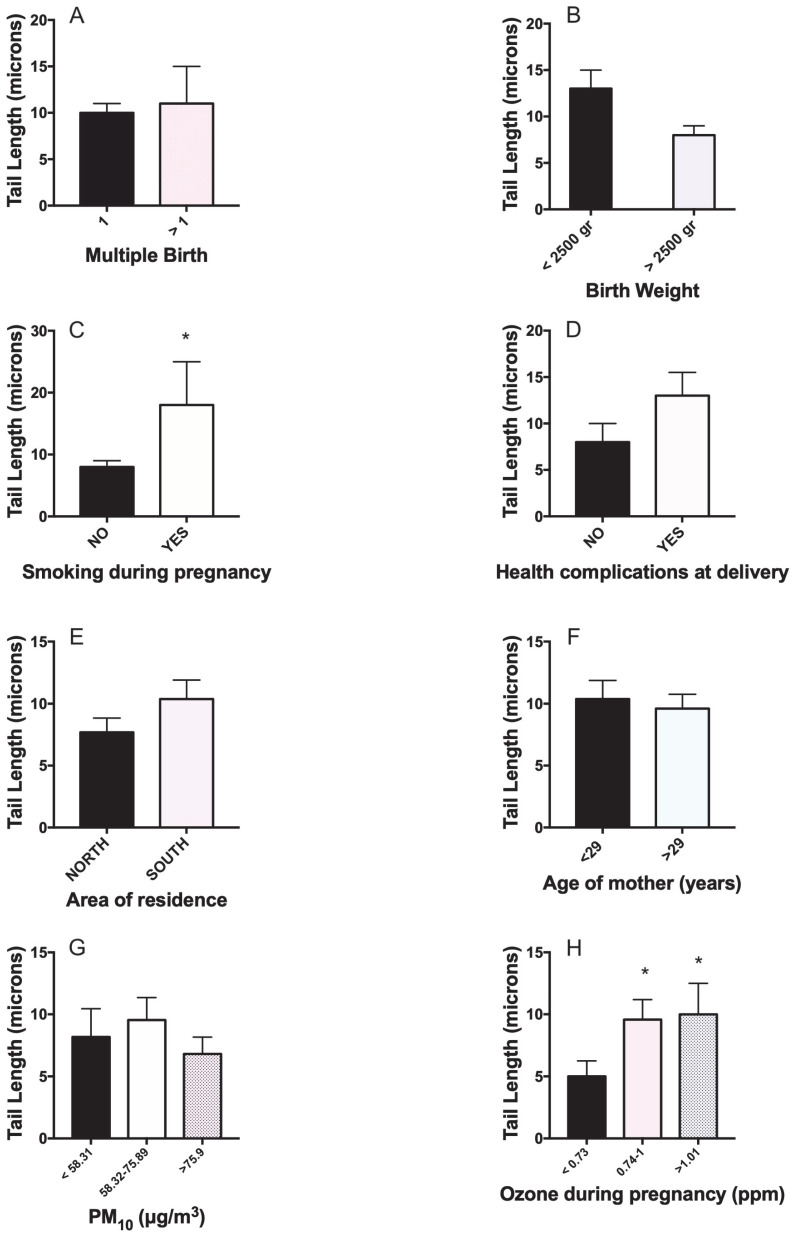
Univariate analysis linking DNA damage (TL) in newborns of Mexico City to main predictive variables. (**A**) multiple births, (**B**) birth weight, (**C**) smoking habit of the mother, (**D**) health complications at delivery, (**E**) area of residence, (**F**) age of mother, (**G**) PM_10_ concentration, (**H**) ozone during pregnancy. * *p* < 0.05.

As far as DNA damage in newborns is concerned (Figure 2A–H), we found only an association with the smoking habits of the mother and with exposure to ozone during pregnancy (Figure 2C,H). However, newborns showing low birth weight or experiencing health complications at delivery reported a borderline increased level of damage (*p* < 0.051) (Figure 2B,D). Interestingly, smoking during pregnancy results in borderline DNA damage increase for mothers, while for newborns, the smoking habit of their mothers means an increase in basal DNA damage (Figure 1C and Figure 2C).

**Figure 3:**
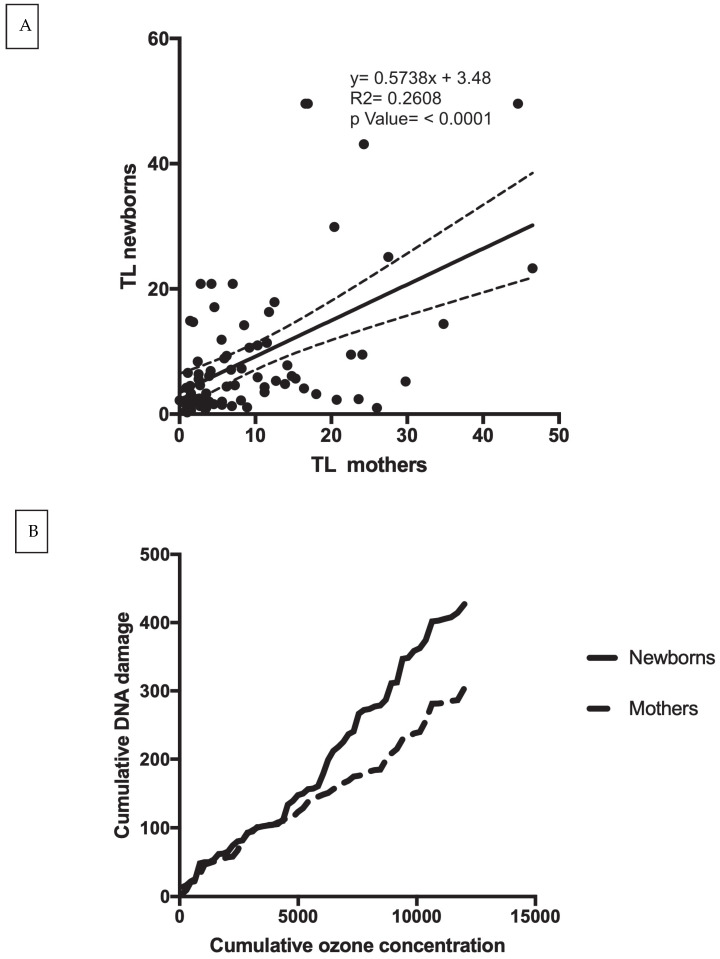
(**A**) Scatter plot and correlation of the level of DNA damage (TL) in mothers vs. DNA damage (TL) in their newborns; (**B**) comparison of accumulated level of DNA damage (TL) with accumulated ozone concentration of both mothers and newborns.

Furthermore, we found a significant correlation between DNA damage in mother–newborn couples (r = 0.511, *p* < 0.001) (Figure 3A). However, we found that the newborns accumulate more damage than the mothers (Figure 3B).

**Figure 4:**
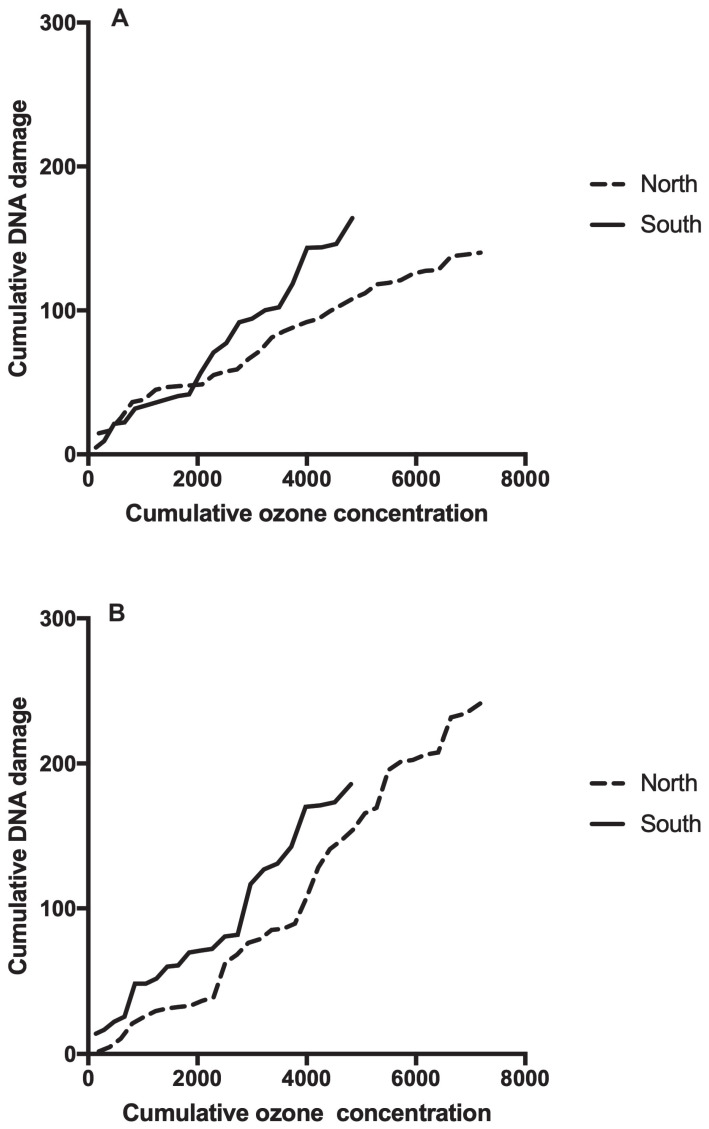
(**A**) Plot of cumulative levels of DNA damage (TL) of mothers living in the southern area of the city compared to mothers living in the northern zone. (**B**) Plot of cumulative levels of DNA damage (TL) of newborns whose mothers lived in the southern part of the city compared to newborns whose mothers live in the northern part of the city.

Mothers who lived in the south of the city (a highly ozone-contaminated area) showed a double level of DNA damage when compared to those who lived in the northern part of the city (TL 8.64 μm (South) vs. 4.18 μm (North), *p* < 0.05) (Figure 4A).

In addition, our hypothesis was partially confirmed in newborns. In them, we observed a 39.3% increase in the DNA damage of newborns whose mothers lived in the southern part of the city compared to mothers who lived in the north; however, this increase was not statistically significant (TL 9.89 μm (South) vs. 7.10 μm (North), *p* = 0.19) (Figure 4B).

To take into account the presence of confounding variables and evaluate the possible interaction between variables in determining DNA damage, separate multivariate lognormal models were fitted to data in the group of mothers and in that of newborns.

The increased level of DNA damage in mothers living in the southern part of Mexico City was confirmed, with a TL 94.0% longer than in mothers living in the northern quarters [MR = 1.94; 95% CI= 1.03–3.63]. The results concerning the role of single pollutants were quite contrasting. While the concentration of ozone at the delivery day significantly increased in the tertile exposed to the highest concentrations, i.e., ≥1.01 ppm, [MR = 3.51; 95% CI = 1.53–8.03], a non-dose-response trend was found for PM_10_ (Table 3).

With respect to NOx, a reduction in DNA damage was observed among mothers exposed to the intermediate tertile of this pollutant [MR = 0.46; 95% CI = 0.24–0.94]. Among other covariates, a significant increase in DNA damage was observed for the mother’s age, with mothers older than 29 years showing a level of damage 2.41 times higher [95% CI 1.04–5.60, *p* < 0.05], and in mothers with health complications at delivery [MR = 2.23; 1.16–4.31; *p* < 0.05] (Table 3). The multivariate analysis of parameters influencing the level of DNA damage in newborns is reported in Table 4. Higher values were found in newborns experiencing health complications at delivery MR = 1.78 [1.02–3.11, *p* < 0.05], in those whose mothers smoked during pregnancy, i.e., MR = 2.72 [1.06–6.93, *p* < 0.05], and in low-birth-weight newborns MR = 1.76 [1.00–3.09, *p* = 0.05].

## 4. Discussion

The harmful effects caused by environmental pollution and associated genotoxic damage in individuals living in highly polluted urban areas have been extensively studied in Mexico. The latest Global Burden of Disease report [9], which considered exposure to air pollutants during pregnancy as a risk to the mother, also extends this risk to newborns. In this report, the risk of respiratory infections; tracheal, bronchial and lung cancer; ischemic processes; and chronic obstructive diseases associated with exposure to PM and ozone during pregnancy was evaluated. That is why we decided to study different variables intrinsically and extrinsically affecting the genetic integrity of newborns in a highly polluted area such as Mexico City. We decided to design a biomonitoring study evaluating DNA damage in exposed mothers and newborns because we were able to identify potential risk variables according to the Global Burden of Diseases report [9]. As a result of in utero exposure, the basal DNA damage in the umbilical cord leukocytes of newborns in Mexico City was measured as a DNA-TL of 9.51 ± 9.57 μm. Surprisingly, the average maternal DNA-TL value was lower (7.74 ± 9.05 μm), especially in comparison with the value observed in our previous report on non-pregnant young women from the same city (12.22 ± 4.38 μm) [29]. This could be due to the fact that women have better nourishment and multivitamin supplementation during pregnancy, which results in increased antioxidant defense [32,33]. In addition, the slightly higher DNA damage of newborns when compared to their mothers is a condition reported previously by other studies comparing DNA damage and other genotoxic endpoints in newborns and adults [34]. This result suggests that newborns had increased susceptibility to environmental genotoxic agents, a condition also reported by other reports in newborns and adults [34]. Additionally, the presence of a correlation between DNA damage in newborns and mothers is known, and it was extensively discussed by Sram et al., who used the comet assay to evaluate DNA damage in the peripheral white blood cells of newborns in the Czech Republic [35]. Thus, the probability of finding DNA damage in the child of a mother with risk factors during pregnancy is higher [36,37,38,39,40].

In agreement with other reports, most of the adverse clinical conditions occurring during pregnancy, such as the number of pregnancies, the number of previously aborted fetuses, and the multiplicity of births, were correlated with an increase in DNA damage in the venous blood cells of mothers [34,41,42].

Our data show that health complications at birth, such as respiratory difficulties, malformations, obstetric trauma, and neuropathies, in addition to nutritional deficiencies, result in a constant but non-significant increase in DNA migration in newborns, as evaluated by the alkaline comet assay.

Smoking during pregnancy increased the level of DNA damage in newborns, confirming results previously reported by other groups [34,43,44,45,46,47,48]. Low birth weight has also been associated with smoking during pregnancy [49]. In our multivariate analysis, these three variables independently explained DNA damage in Mexico City newborns, i.e., health complications at delivery, low birth weight, and smoking during pregnancy. It is important to mention that even when we found a tendency of DNA damage increase in relationship with PM_10_ exposure, this could be related to low birth weight, as other studies mention [50]. Trying to further assess the potential interaction of these two air pollutants on inducing DNA damage, we performed an additive model. We used the synergy index [51,52]; however, we did not find any additive effects between ozone and PM_10_ concentrations.

In this work, we were able to substantiate, in a different timeframe, our previous results, where we observed that mothers living in the southern part of the city present greater DNA damage [28], even though current results present a lower average value than in our previous work. On the other hand, DNA damage in newborns also tends to be greater when mothers lived in the southern area of Mexico City, although results did not reach statistical significance, possibly because of additional heterogeneity due to a cluster of children whose mothers lived in the northwestern part of the city. It is worth mentioning that this increase is not related to any of the pollutants evaluated; however, a random clustering due to the small number of subjects (n = 7) cannot be ruled out.

In addition to the fact that newborns’ DNA damage correlates with the accumulation of ozone during pregnancy, the results of the present study suggest the possibility that protection from the other pollutants present in the southern area of the city can be offered by the placenta. Interestingly, mothers living in the northern area had the lowest DNA damage, and this area showed the lowest concentrations of ozone [26,27,29,30,53,54], opposite to the highest concentration of NOx. In this regard, it has recently been reported that NOx can generate inter-crosslinks due to the formation of a double-strand diazonium ion intermediate, and this could explain the reduction in DNA migration that we observed in mothers who are exposed to high concentrations of NOx [55]. It is worth noting that this kind of damage can drastically affect the structure and function of DNA.

The slightly higher DNA damage that we observed in newborns can be explained by the existing report of less nucleotide excision repair and fewer antioxidant abilities in newborns compared to mothers [56,57]. It is important to mention that there are many factors that can affect the estimation of personal exposure to pollutants, such as the time spent indoors [58], the type of building [59], the air filtration system [60], the occupancy [61], and the interior sources (such as cooking) [62]. In our study, we found that most of the mothers’ work and workplaces are very close to their place of residence and that all mothers lived inside Mexico City, and we did not observe differences between the time they spent working, so the monitor closest to their place of residence was assigned to each mother. However, we believe that in future works, it will be necessary to use personalized monitors for a more accurate estimate of exposure to these environmental contaminants.

## 5. Conclusions

Basal DNA damage in both newborns and their mothers, as well as the in utero exposure history, could be determined by the comet assay. The sensibility of the test is sufficiently strong to identify differentially the effects of the exposure during pregnancy by mother–newborn couples, and it allows for the estimation of long-term risks [63]. The identification of variables related to DNA damage both in newborns (health complications in childbirth, smoking habits of mothers during pregnancy, low birth weight, and ozone during pregnancy) and mothers (maternal age > 29 years, health complications in childbirth, living in the southern area of the city, high ozone level at 1 ppm, and mid-range exposure to NOx and PM_10_) indicates that environmental pollution can be considered a risk starting from in utero exposure, and this could have long-term repercussions in newborns [64]. Early control of these exposures may help to reduce the long-term effects caused by in utero exposures. These conditions can have an impact on health later in life, including serious conditions such as childhood cancers and immune disorders. To improve prevention strategies of these disorders, additional research should be planned to assess DNA damage as a marker of risk in exposome studies. Prospective studies are required to validate biomarkers such as the comet assay as suitable tools for disease prevention in public health.

## Figures and Tables

**Table 1:** Descriptive analysis of study groups, mother residents in Mexico City and their newborns.

Mother/Newborn/Delivery Characteristics		N	Mean ± SD or %	Min-Max
Maternal age (years)		79	28.5 ± 6.3	14–43
Multiple births	1	94	90.4%	1–4
	>1	10	9.6%	
Number of pregnancies	1	14	30.4%	1–7
	>1	32	69.6%	
Number of abortions	No	33	68.8%	0–4
	Yes	15	31.3%	
Infections during pregnancy	No	71	73.2%	
	Yes	26	26.8%	
Use of medications during pregnancy	No	70	72.2%	
	Yes	27	27.8%	
Smoking during pregnancy	No	39	86.7%	0–20 (Cigarettes/Day)
	Yes	6	13.3%	
Weeks of gestational age	≥37	47	54.0%	27–42 (Weeks)
	<37	40	46.0%	
Birth weight (%)	≥2500 g	58	76.7%	1114–4500 (g)
	<2500 g	24	29.3%	
Apgar score at 1 min		78	7.99 ± 1.15	1–9
Apgar score at 5 min		80	8.81 ± 0.53	6–9
Area of residence (2 levels)	North	42	58.9%	
	South	31	41.1%	
Area of residence (4 levels)	North-West	7	9.7%	
	North-East	35	47.9%	
	South-West	19	26.0%	
	South-East	12	16.4%	

SD: standard deviation, g: grams.

**Table 2:** The average concentration of NOx, PM_10_, and ozone in the group of mothers living in Mexico City. Exposure to ozone was calculated as: (1) average concentration of the first trimester; (2) average concentration during pregnancy; (3) ozone concentration on the day of delivery.

Exposure to Air Pollution	N	Mean ± SD	Min-Max
NOx (ppm)	70	1.32 ± 0.35	0.425–2.72
PM_10_ (μg/m^3^)	52	69.25 ± 10.48	40.75–93.46
Ozone (ppm)	52	0.81 ± 0.10	0.51–1.90
Ozone 1st trimester (ppm)	70	0.89 ± 0.18	0.315–1.26
Ozone at delivery (ppm)	70	0.85 ±0.34	0.33–1.87

SD: standard deviation.

**Table 3:** Multivariate lognormal modelling of the association between selected predictive variables and DNA damage (TL) in mothers.

Variables		β ± SD	MR [95% CI]	*p*-Value
Area of Residence	NorthSouth	-	1.00	<0.05
		0.66 ± 0.31	1.94; [1.03–3.63]	
NOx (ppm)	≤1.19	-	1.00	
	1.20–1.96	−0.77 ± 0.36	0.46; [0.24–0.94]	<0.05
	≥1.97	0.72 ± 0.41	0.38; [0.21–1.12]	n.s.
PM_10_ (μg/m^3^)	≤58.31	-	1.00	
	58.32–75.89	−0.75 ± 0.37	0.47; [0.23–0.99]	≤0.05
	≥75.90	−0.50 ± 0.37	0.61; [0.19–0.78]	≤0.05
Ozone at delivery (ppm)	≤0.73	-	1.00	
	0.74–1.00	0.06 ± 0.32	1.06; [0.56–2.01]	n.s.
	≥1.01	1.26 ± 0.41	3.51; [1.53–8.03]	<0.01
Maternal age	<29	-	1.00	<0.05
	≥29	0.88 ± 0.41	2.41; [1.04–5.60]	
Difficult delivery	No	-	1.00	<0.05
	Yes	0.80 ± 0.33	2.23; [1.16–4.31]	

SD: standard deviation.

**Table 4:** Multivariate lognormal modelling of the association between selected predictive variables and DNA damage (TL) in newborns.

Variables		β ± SD	MR [95% CI]	*p*-Value
Difficult delivery	No Yes	-	1.00	<0.05
		0.58 ± 0.28	1.78; [1.02–3.11]	
Birth weight	≥2500 g	-	1.00	
	<2500 g	0.57 ± 0.28	1.76; [1.00–3.09]	<0.05
Smoking during pregnancy	No	-	1.00	<0.05
	Yes	0.99 ± 0.46	2.72; [1.06–6.93]	

SD—standard deviation.

## Data Availability

Data available on request due to restrictions. The data presented in this study are available on request from the corresponding author. The data are not publicly available due to privacy restrictions.

## References

[1] Dondi A., Carbone C., Manieri E., Zama D., Del Bono C., Betti L., Biagi C., Lanari M. (2023). Outdoor Air Pollution and Childhood Respiratory Disease: The Role of Oxidative Stress. Int. J. Mol. Sci..

[2] Molina L.T., Velasco E., Retama A., Zavala M. (2019). Experience from Integrated Air Quality Management in the Mexico City Metropolitan Area and Singapore. Atmosphere.

[3] Mamkhezri J., Bohara A.K., Islas Camargo A. (2020). Air pollution and daily mortality in the Mexico City Metropolitan Area. Atmósfera.

[4] Texcalac-Sangrador J.L., Hurtado-Díaz M., Félix-Arellano E.E., Guerrero-López C.M., Riojas-Rodríguez H. (2021). Health and Economic Impacts Assessment of O_3_ Exposure in Mexico. Int. J. Environ. Res. Public Health.

[5] Cerón R.M., Cerón J.G., Rangel M., Ruíz A., Aguilar C., Montalvo C., Canedo Y., García R., Uc M., Galván A. (2023). Association between Short-Term Exposure to Criteria Air Pollutants and Daily Mortality in Mexico City: A Time Series Study. Atmosphere.

[6] Gutiérrez-Avila I., Riojas-Rodríguez H., Colicino E., Rush J., Tamayo-Ortiz M., Borja-Aburto V.H., Just A.C. (2023). Daily exposure to PM_2.5_ and 1.5 million deaths: A time-stratified case-crossover analysis in the Mexico City Metropolitan Area. medRxiv.

[7] Calderón-Garcidueñas L., Ayala A. (2023). Fine particle air pollution and lung cancer risk: Extending the long list of health risks. Cell.

[8] Romieu I., Gouveia N., Cifuentes L.A., de Leon A.P., Junger W., Vera J., Strappa V., Hurtado-Díaz M., Miranda-Soberanis V., Rojas-Bracho L. (2012). Multicity study of air pollution and mortality in Latin America (the ESCALA study). Res. Rep. Health Eff. Inst..

[9] GBD 2019 Chronic Respiratory Diseases Collaborators (2023). Global burden of chronic respiratory diseases and risk factors, 1990–2019: An update from the Global Burden of Disease Study 2019. EClinicalMedicine.

[10] Tellez-Rojo M.M., Romieu I., Polo-Pena M., Ruiz-Velasco S., Meneses-Gonzalez F., Hernandez-Avila M. (1997). Effect of environmental pollution on medical visits for respiratory infections in children in Mexico City. Salud Publica Mex..

[11] Pereira L.A., Loomis D., Conceicao G.M., Braga A.L.F., Arcas R.M., Kishi H.S., Singer J.M., Bohm G.M., Saldiva P.H. (1998). Association between air pollution and intrauterine mortality in Sao Paulo, Brazil. Environ. Health Perspect..

[12] Davis D.L., Saldiva P.H.N. (1999). Urban Air Pollution Risks to Children: A Global Environmental Health Indicator. http://www.wri.org/pubs/pubs_description.cfm?pid=3004.

[13] Loomis D., Castillejos M., Gold D.R., McDonnell W., Borja-Aburto V.H. (1999). Air pollution and infant mortality in Mexico City. Epidemiology.

[14] Yang Y., Runkui L., Wenjing L., Wang M., Cao Y., Wu Z., Xu Q. (2013). The Association between Ambient Air Pollution and Daily Mortality in Beijing after the 2008 Olympics: A Time Series Study. PLoS ONE.

[15] Torrico-Lavayen R., Vargas-Alarcón G., Riojas-Rodriguez H., Sánchez Guerra M.A., Texcalac-Sangrador J.L., Ortiz-Panozo E., Gutiérrez-Avila I., De Vizcaya-Ruiz A., Cardenas A., Posadas-Sánchez R. (2023). Long-term exposure to ambient fine particulate matter and carotid intima media thickness at bilateral, left and right in adults from Mexico City: Results from GEA study. Chemosphere.

[16] Rojas-Martinez R., Perez-Padilla R., Olaiz-Fernandez G., Mendoza-Alvarado L., Moreno-Macias H., Fortoul T., Mc Donnell W., Loomis D., Romieu I. (2007). Lung function growth in children with long-term exposure to air pollutants in Mexico City. Am. J. Respir. Crit. Care Med..

[17] Escamilla-Nunez M.C., Barraza-Villareal A., Hernandez-Cadena L., Moreno-Macias H., Ramirez-Aguilar M., Sienra-Monje J.J., Cortez-Luga M., Texcalac J.L., del Rio-Navarro B., Romieu I. (2008). Traffic-related air pollution and respiratory symptoms among asthmatic children, resident in Mexico City: The EVA cohort study. Respir. Res..

[18] Barraza-Villarreal A., Sunyer J., Hernandez-Cadena L., Escamilla Nunez M.C., Sienra-Monje J.J., Ramirez-Aguilar M., Cortez-Lugo M., Holguin F., Diaz-Sanchez D., Olin A.C. (2008). Air pollution, airway inflammation, and lung function in a cohort Study of Mexico City Schoolchildren. Environ. Health Perspect..

[19] Barraza-Villarreal A., Escamilla-Nunez M.C., Hernandez-Cadena L., Texcalac-Sangrador J.L., Sienra-Monje J.J., del Rio-Navarro B.E., Cortez-Lugo M., Sly P.D., Romieu I. (2011). Elemental carbon exposure and lung function in schoolchildren from Mexico City. Eur. Respir. J..

[20] Linares B., Guizar J.M., Amador N., Garcia A., Miranda V., Perez J.R., Chapela R. (2010). Impact of air pollution on pulmonary function and respiratory symptoms in children. Longitudinal repeated-measures study. BMC Pulm. Med..

[21] Li P., Xin J., Wang Y., Li G., Pan X., Wang S., Cheng M., Wen T., Wang G., Liu Z. (2014). Association between particulate matter and its chemical constituents of urban air pollution and daily mortality or morbidity in Beijing City. Environ. Sci. Pollut. Res..

[22] Pedersen M., Mendez M.A., Schoket B., Godschalk R.W., Espinosa A., Landström A., Villanueva C.M., Merlo D.F., Fthenou E., Gracia-Lavedan E. (2015). Environmental, dietary, maternal, and fetal predictors of bulky DNA adducts in cord blood: A European mother–child study (NewGeneris). Environ. Health Perspect..

[23] Valverde M., Rojas E., Dhawan A., Anderson D. (2017). Chapter 11: Comet Assay in Human Biomonitoring. Issues in Toxicology.

[24] Singh N.P., McCoy M.T., Tice R.R., Schneider E.L. (1988). A simple technique for quantitation of low levels of DNA damage in individual cells. Exp. Cell Res..

[25] Collins A., Møller P., Gajski G., Vodenková S., Abdulwahed A., Anderson D., Bankoglu E.E., Bonassi S., Boutet-Robinet E., Brunborg G. (2023). Measuring DNA modifications with the comet assay: A compendium of protocols. Nat. Protoc..

[26] Calderon-Garciduenas L., Osnaya-Brizuela N., Ramirez-Martinez L., Villarreal-Calderon A. (1996). DNA Strand Breaks in Human Nasal Respiratory Epithelium Are Induced upon Exposure to Urban Pollution. Environ. Health Perspect..

[27] Calderón-Garcidueñas L., Osnaya N., Rodríguez-Alcaraz A., Villarreal-Calderón A. (1997). DNA damage in nasal respiratory epithelium from children exposed to urban pollution. Environ. Mol. Mutagen..

[28] Tovalin H., Valverde M., Morandi M.T., Blanco S., Whitehead L., Rojas E. (2006). DNA damage in outdoor workers occupationally exposed to environmental air pollutants. Occup. Environ. Med..

[29] Valverde M., López M.C., López I., Sánchez I., Fortoul T.I., Ostrosky-Wegman P., Rojas E. (1997). DNA damage in leukocytes and buccal and nasal epithelial cells of individuals exposed to air pollution in Mexico City. Environ. Mol. Mutagen..

[30] Rojas E., Valverde M., López M.C., Naufal I., Bizarro P., López I., Fortoul T.I., Ostrosky-Wegman P. (2000). Evaluation of DNA damage in exfoliated tear duct epithelial cells from individuals exposed to air pollution assessed by single cell gel electrophoresis assay. Mutat. Res. Genet. Toxicol. Environ. Mutagen..

[31] Tice R.R., Agurell E., Burlinson B., Hartmann A., Kobayashi H., Miyame Y., Rojas E., Ryu J.C., Sasaki Y.F. (2000). Single cell gel/comet assay: Guidelines for in vitro and in vivo genetic toxicology testing. Environ. Mol. Mutagen..

[32] Scalera F., Fischer T., Schlembach D., Beinder E. (2002). Serum from healthy pregnant women reduces oxidative stress in human umbilical vein endothelial cells. Clin. Sci..

[33] Baydas G., Karatas F., Gursu M.F., Bozkurt H.A., Ilhan N., Yasar A., Canatan H. (2002). Antioxidant vitamin levels in term and preterm infants and their relation to maternal vitamin status. Arch. Med. Res..

[34] Neri M., Ugolini D., Bonassi S., Ficic A., Holland N., Knudsen L.E., Sram R.J., Ceppi M., Bocchini V., Merlo D.F. (2006). Children’s exposure to environmental pollutants and biomarkers of genetic damage: II. Results of a comprehensive literature search and meta-analysis. Mutat. Res. Rev. Mutat. Res..

[35] Šrám R.J., Podrazilová K., Dejmek J., Mračková G., Pilčík T. (1998). Single cell gel electrophoresis assay: Sensitivity of peripheral white blood cells in human population studies. Mutagenesis.

[36] Whyatt R.M., Perera F.P. (1995). Application of biologic markers to studies of environmental risks in children and the developing fetus. Environ. Health Perspect..

[37] Dussias V., Stefos T., Stefanidis K., Paraskevaidis E., Karabini F., Lolis D. (1997). Lead concentrations in maternal and umbilical cord blood in areas with high and low air pollution. Clin. Exp. Obstet. Gynecol..

[38] Whyatt R.M., Santella R.M., Jadrychowsky W., Garte S.J., Bell D.A., Ottman R., Gladek-Yarborough A., Cosma G., Young T.L., Cooper T.B. (1998). Relationship between ambient air pollution and DNA damage in Polish mothers and newborns. Environ. Health Perspect..

[39] Binkova B., Veselyý D., Veselá D., Jelínek R., Sram R.J. (1999). Genotoxicity and embryotoxicity of urban air particulate matter collected during winter and summer period in two different districts of the Czech Republic. Mutat. Res. Genet. Toxicol. Environ. Mutagen..

[40] Dejmek J., Selevan S.G., Benes I., Solansky I., Sram R.J. (1999). Fetal growth and maternal exposure to particulate matter during pregnancy. Environ. Health Perspect..

[41] Mattison D.R. (2010). Environmental exposures and development. Curr. Opin. Pediatr..

[42] Furnees D.L., Dekker G.A., Roberts C.T. (2011). DNA damage and health in pregnancy. J. Reprod. Immunol..

[43] Sardas S., Karahatil B., Akyol D., Kukner S., Karakaya A.E. (1995). Assessment of smoking-induced DNA damage in lymphocytes of smoking mothers of newborn infants using the alkaline single-cell gel electrophoresis technique. Mutat. Res. Mutagen. Relat. Subj..

[44] Sardas S., Walker D., Akyol D., Karakaya A.E. (1995). The effect of smoking on sister chromatid exchange rate of newborn infants born to smoking mothers. Mutat. Res. Toxicol..

[45] Betancourt M., Ortiz R., Gonzalez C., Perez P., Cortes L., Rodriguez L., Villasenor L. (1995). Assessment of DNA damage in leukocytes from infected and malnourished children by single cell gel electrophoresis/comet assay. Mutat. Res. Fundam. Mol. Mech. Mutagen..

[46] Perera F.P., Whyat R.M., Rauh V., Whyatt R.M. (1998). 1 Recent developments in molecular, epidemiology: A study of the effects of environmental polycyclic aromatic hydrocarbons on birth outcomes in Poland. Am. J. Epidemiol..

[47] Perera F.P., Whyatt R.M., Jedrychowski W., Rauh V., Manchester D., Santella R.M., Ottman R. (1999). Molecular epidemiologic research on the effects of environmental pollutants on the fetus. Environ. Health Perspect..

[48] de Assis K.R.C., Ladeira M.S.P., Bueno R.C.A., dos Santos B.F., Dalben I., Salvadori D.M.F. (2009). Genotoxicity of cigarette smoking in maternal and newborn lymphocytes. Mutat. Res. Genet. Toxicol. Environ. Mutagen..

[49] Chelchowska M., Ambroszkiewicz J., Gajewska J., Laskowska-Klita T., Leibschang J. (2011). The effect of tobacco smoking during pregnancy on plasma oxidant and antioxidant status in mother and newborn. Eur. J. Obstet. Gynecol. Reprod. Biol..

[50] Xu X., Sharma R.K., Talbott E.O., Zborowski J.V., Rager J., Arena V.C., Volz C.D. (2011). PM10 air pollution exposure during pregnancy and term low birth weight in Allegheny County, PA, 1994–2000. Int. Arch. Occup. Environ. Health.

[51] Anderson T., Alfredsson L., Kälberg H., Zdravkovic S., Ahlbom A. (2005). Calculating measures of biological interaction. Eur. J. Epidemiol..

[52] Ruan Z., Qian Z.M., Guo Y., Zhou J., Yang Y., Acharya B.K., Guo S., Zheng Y., Cummings-Vaughn L.A., Rigdon S.E. (2019). Ambient fine particulate matter and ozone higher that certain thresholds associated with myopia in the elderly aged 50 years and above. Environ. Res..

[53] Borja-Aburto V.H., Castillejos M., Gold D.R., Bierzwinski S., Loomis D. (1998). Mortality and ambient fine particles in southwest Mexico City, 1993–1995. Environ. Health Perspect..

[54] Rojas E., López M.C., Valverde M. (1999). Single cell gel electrophoresis assay: Methodology and applications. J. Chromatogr. B Biomed. Sci. Appl..

[55] Hernandez-Haro N., Solis-Calero C., Casasnovas R., Morell C., Grand A., Frau J., Ortega-Castro J. (2022). Formation mechanism of Inter-Crosslink in DNA by nitrogen oxides pollutants through a diazonium intermediate. Int. J. Mol. Sci..

[56] Vande Loock K., Decordier I., Plas G., Ciardelli R., Haumont D., Kirsch-Volders M. (2014). Lower nucleotide excision repair capacity in newborns compared to their mothers: A pilot study. Reprod. Toxicol..

[57] Mesbah-Namin S.A., Shahidi M., Nakhshab M. (2017). An increased genotoxic risk in lymphocytes from phototherapy-treated hyperbilirubinemic neonates. Iran. Biomed. J..

[58] Nazaroff W.W., Weschler C.J. (2022). Indoor ozone: Concentrations and influencing factors. Indoor Air.

[59] Chen C., Zhao B. (2011). Review of relationship between indoor and outdoor particles: I/O ratio, infiltration factor and penetration factor. Atmos. Environ..

[60] Wang P., Liu J., Wang C., Zhang Z., Li J. (2022). A holistic performance assessment of duct-type electrostatic precipitators. J. Clean. Prod..

[61] Li J., Zuraimi S., Schiavon S., Wan M.P., Xiong J., Tham K.W. (2022). Diurnal trends of indoor and outdoor fluorescent biological aerosol particles in a tropical urban area. Sci. Total Environ..

[62] Wang P., Liu S., Liu J., Wang J., Li J. (2022). Size-resolved splashed cooking oil droplets from 1 to 1000 μm on surfaces: The impact of residential range hoods. Build. Environ..

[63] Bonassi S., Ceppi M., Møller P., Azqueta A., Milić M., Neri M., Brunborg G., Godschalk R., Koppen G., Langie S.A.S. (2021). DNA damage in circulating leukocytes measured with the comet assay may predict the risk of death. Sci. Rep..

[64] Melody S.M., Ford J., Wills K., Venn A., Johnston F.H. (2019). Maternal exposure to short to medium-term outdoor air pollution and obstetric and neonatal outcomes: A systematic review. Environ. Pollut..

